# Supplementary material for: The genome sequence of the Camberwell Beauty, Nymphalis antiopa (Linnaeus, 1758) (Lepidoptera: Nymphalidae)
Source: Wellcome Open Res. 2025 Aug 11;10:421. [Version 1] doi: 10.12688/wellcomeopenres.24676.1 (PMC12464534; doi:10.12688/wellcomeopenres.24676.1)
Supplement: Peer review history

# Reviewer report: Reviewer response for version 1

**DOI:** 10.21956/wellcomeopenres.27191.r132661

**Published:** 2025-10-08

**Authors:** Panagiotis Ioannidis

**Affiliations:** 1 Foundation for Research & Technology - Hellas, Crete, Greece

This paper describes the genome sequencing and assembly of the lepidopteran Nymphalis antiopa.

 The standard methodology the authors used resulted in a high quality genome assembly, as shown by all quality metrics.

 I particularly like the Background part where the authors provided details about the genomic resources currently available for this species, as well as a brief statement about how the herein reported genome assembly could shed light in the evolution of Lepidoptera. I also liked how the authors included a figure on Merian elements (figure 3).

Are sufficient details of methods and materials provided to allow replication by others?

Yes

Is the rationale for creating the dataset(s) clearly described?

Yes

Are the datasets clearly presented in a useable and accessible format?

Yes

Are the protocols appropriate and is the work technically sound?

Yes

Reviewer Expertise:

insect genomics; bioinformatics

I confirm that I have read this submission and believe that I have an appropriate level of expertise to confirm that it is of an acceptable scientific standard.

# Reviewer report: Reviewer response for version 1

**DOI:** 10.21956/wellcomeopenres.27191.r132659

**Published:** 2025-09-25

**Authors:** Arun Arumugaperumal

**Affiliations:** 1 Department of Biotechnology, Rajalakshmi Engineering College, Thandalam 602105, Chennai, Tamil Nadu, India

The data note describes the genome sequence of a butterfly species named *Nymphalis antiopa*. The authors have clearly documented the photograph of the specimen in Figure 1. It will be very helpful for the readers to recognize the butterfly species. This is the first report of the high-quality genome sequence of the butterfly species. The authors have used advanced sequencing technology to obtain the genome sequence of *Nymphalis antiopa*. The assembly reported here is of size 392.57 Mb, spread among 31 chromosomes, including the Z sex chromosome. The collected specimen was a male. The genome of another Nymphalis genus member, a female *N. antiopa,* was reported to have ZW sex chromosomes. Hence, the authors could have chosen a female butterfly to get the sequence of the W chromosome. A BUSCO score of 98.7%  completeness indicates that the genome sequence is a near-complete one. The genome is yet to be annotated through Ensembl. The accession IDs and links are in working condition. The article can be indexed.

Are sufficient details of methods and materials provided to allow replication by others?

Yes

Is the rationale for creating the dataset(s) clearly described?

Yes

Are the datasets clearly presented in a useable and accessible format?

Yes

Are the protocols appropriate and is the work technically sound?

Yes

Reviewer Expertise:

Genomics; Bioinformatics

I confirm that I have read this submission and believe that I have an appropriate level of expertise to confirm that it is of an acceptable scientific standard.

# Reviewer report: Reviewer response for version 1

**DOI:** 10.21956/wellcomeopenres.27191.r132660

**Published:** 2025-09-25

**Authors:** Leonardo Dapporto

**Affiliations:** 1 Università di Firenze, Florence, Italy

In this article the authors present a genome assembly from a specimen of *Nymphalis antiopa*. To the extent of my ability to evaluate genome quality, the sequencing and assembly appear to be of high standard, consistent with other assemblies produced within the Psyche project.

 However, the sequenced specimen was a male, which means that the assembly includes the Z chromosome but not the W. It would be useful if the authors clarified this point and explained why a male was chosen, since this may not be obvious to all readers. Given that the species is often common, one might expect that a female specimen could also have been available to investigate the W chromosome.

 I do not see other major issues with the manuscript. It might be worth mentioning that analyses of BOLD data show that North American and Palearctic populations are separated by only a few COI mutations and are both characterised by low genetic differentiation, as reported by Dapporto et al. (2022, already cited) and D’Ercole et al. (2024) [Reference 1]

Are sufficient details of methods and materials provided to allow replication by others?

Yes

Is the rationale for creating the dataset(s) clearly described?

Yes

Are the datasets clearly presented in a useable and accessible format?

Yes

Are the protocols appropriate and is the work technically sound?

Yes

Reviewer Expertise:

Comparative hylogeography, taxonomy, ecology

I confirm that I have read this submission and believe that I have an appropriate level of expertise to confirm that it is of an acceptable scientific standard.

## References

[1] : A genetic atlas for the butterflies of continental Canada and United States. *PLOS ONE* .2024;19(4) : 10.1371/journal.pone.0300811 10.1371/journal.pone.0300811 PMC1099019938568891
